# Yeast-based directed-evolution for high-throughput structural stabilization of G protein-coupled receptors (GPCRs)

**DOI:** 10.1038/s41598-022-12731-2

**Published:** 2022-05-23

**Authors:** M. Meltzer, T. Zvagelsky, U. Hadad, Niv Papo, Stanislav Engel

**Affiliations:** 1 Department of Clinical Biochemistry and Pharmacology, Faculty of Health Sciences, Ben-Gurion University of the Negev, P.O.B. 653, Beer-Sheva, 8410501 Israel; 2 Ilse Katz Institute for Nanoscale Science and Technology, Ben-Gurion University of the Negev, Beer-Sheva, Israel; 3 Avram and Stella Goldstein-Goren Department of Biotechnology Engineering, Faculty of Engineering, Ben-Gurion University of the Negev, P.O.B. 653, Beer-Sheva, 8410501 Israel; 4 National Institute for Biotechnology in the Negev, Ben-Gurion University of the Negev, Beer-Sheva, Israel

**Keywords:** X-ray crystallography, G protein-coupled receptors, Membrane proteins, Screening

## Abstract

The immense potential of G protein-coupled receptors (GPCRs) as targets for drug discovery is not fully realized due to the enormous difficulties associated with structure elucidation of these profoundly unstable membrane proteins. The existing methods of GPCR stability-engineering are cumbersome and low-throughput; in addition, the scope of GPCRs that could benefit from these techniques is limited. Here, we present a yeast-based screening platform for a single-step isolation of GRCR variants stable in the presence of short-chain detergents, a feature essential for their successful crystallization using vapor diffusion method. The yeast detergent-resistant cell wall presents a unique opportunity for compartmentalization, to physically link the receptor's phenotype to its encoding DNA, and thus enable discovery of stable GPCR variants with unprecedent efficiency. The scope of mutations identified by the method reveals a surprising amenability of the GPCR scaffold to stabilization, and suggests an intriguing possibility of amending the stability properties of GPCR by varying the structural status of the C-terminus.

## Introduction

Being the largest protein family (~ 800 members), G protein coupled receptors (GPCRs) are the principal conduits of information from the extracellular matrix to cytosol. Dysregulation and malfunctions of approximately 100 GPCRs are known to associate with various human diseases^1^, including inflammation, infertility, metabolic and neurological disorders, viral infections and cancer^2–5^. Because of their druggability, i.e. therapeutic effect achieved by binding of small-molecule compounds, and membrane localization that facilitates their accessibility to drugs, GPCRs represent a target of upmost importance in contemporary drug discovery^6^.

Structure-based drug design (SBDD), in which the design of new drugs is guided by structural data from protein–ligand complexes, remains one of the most efficient approaches in modern drug discovery^7^. In GPCR, however, the SBDD is hampered by the paucity of structural data due to the notorious difficulties associated with the expression, isolation (in a functional form) and structural elucidation of these profoundly unstable proteins. Currently, less than a hundred unique GPCR structures, solved by either X-ray crystallography or cryo-electron microscopy (cryo-EM), are available at the PDB, while the vast majority of GPCRs, some of potential importance to drug discovery, have not yet been structurally characterized^8^.

Although significant progress has been achieved in GPCR structural determination using cryo-EM, X-ray crystallography remains the best-suited approach for high-throughput structural elucidation typical of SBDD projects, which due to their iterative character may require hundreds of structures to be solved^9^. Structure determination and model construction using cryo-EM may take weeks, compared to hours using X-ray crystallographic data, in which a molecular replacement could be used to expedite the determination of receptor structures bound to different ligands^9,10^. In addition, the resolution of the cryo-EM GPCR structures is typically between 3.0 and 3.5 Å, whereas the local resolution can vary greatly within different regions of the map, and it is typically better at the GPCR-G protein interfaces and worse around the orthosteric ligand-binding pockets^8,9,11^, the latter of main interest for SBDD.

The quality of the sample remains the most critical factor in high-resolution structural determination by both X-ray crystallography and cryo-EM^9^. The number of cryo-EM images required and the resolution achieved correlate with sample quality and benefit significantly from protein stabilization and removal of flexible loops^9,12^. As a consequence, most cryo-EM high-resolution GPCR structures were obtained in the presence of accessory proteins, such as nanobodies or modified G proteins, playing stabilizing role^11,13,14^. Thus, structural engineering of GPCR to improve their stability and thus sample quality may benefit GPCR structural elucidation by both X-ray crystallography and cryo-EM^8^.

Although the main technique of GPCR crystallization remains lipidic cubic phase (LCP), vapor diffusion crystallography—because of its simplicity and amenability to automatization—holds great potential for SBDD, which requires a large number of structures to be determined^15^. In contrast to LCP, in which a receptor, after extraction with a detergent, is reconstituted into lipid bilayer, in vapor diffusion method the crystallization takes place directly in detergent micelles^16^. Detergents with long alkyl chains (> C12) such as *n*-dodecyl β-d-maltoside (DDM), which produce large micelles and thus mimic the membrane environment relatively well, have been successfully used for GPCR isolation in a functional form^17^. The long-chain detergents, however, occlude a significant portion of the hydrophilic surfaces forming lattice contacts in membrane protein crystals, interfering with crystallization. In contrast, detergents with short alkyl chains (< C9), such as *n*-octyl-β-d-glucoside (OG), whose micelles are small, leave a significant portion of the protein's hydrophilic surfaces exposed, thus favoring crystallization. Unfortunately, excessive exposure to solvent combined with a highly dynamic nature of short-chain detergents resulting in their penetration into the core of the transmembrane domain^18^, are destabilizing to receptor's structure. Such incompatibilities could be reconciled through GPCR engineering to improve its structural stability in the presence of short-chain detergents.

Over the years, the methods of GPCR stability engineering have evolved from an alanine-scan mutagenesis of *E. coli*-expressed receptors to improve thermostability^19–23^, to a directed evolution in *E. coli*^24^ or yeast^25^ to increase the level of receptor expression. The latter approach assumes correlation between the level of plasma membrane expression of properly folded receptors and their structural stability. This assumption, however, is not inclusive, and expression-guided sorting may only increase the *probability* of identifying stable variants^20^, failing to reveal the entire sequence space associated with receptor stability present in the library. To overcome the limitation of expression-guided sorting, a technique called CHESS, in which clone selection was actuated directly by the receptor's stability properties, was developed^24,26^. In the CHESS, bacterial cells expressing a library of GPCR variants are chemically coated with a synthetic polymer to yield detergent-resistant capsules. By treating the capsules with a short-chain detergent, membrane proteins are solubilized, and due to their ability to maintain binding of a fluorescent ligand, detergent-resistant variants are isolated by library sorting using FACS^24^. The major drawback of the CHESS method, besides the cumbersome character of the encapsulation procedure, is a limited scope of GPCRs amenable to engineering. In fact, bacteria is a notoriously inefficient host for GPCR production, and up to date only few GPCRs have been successfully expressed in *E. coli*^27^. Moreover, without the posttranslational modifications (PTM) critical to receptors' functioning^28–30^ and absent in prokaryotes, there is a possibility that structural stabilization would trap the  *E. coli*-expressed receptor in an unnatural conformation; drug screening against such target may select compounds, whose behavior toward the native receptor under the physiological conditions (in either potency, efficacy or selectivity) would be unpredictable.

To overcome the drawbacks of the existing approaches and provide effective solution for the growing needs of drug discovery, we devised a yeast-based single-step directed-evolution methodology for high-throughput isolation of GPCR variants stable in the presence of short-chain detergents. The method takes advantage of the yeast’s natural cell wall to link the receptor's phenotype (resistance to detergent) to its genotype (mutation(s) responsible for the phenotype). The proposed methodology is expected to benefit the field of GPCR drug discovery by facilitating the development of receptor variants amenable to high-throughput structural elucidation by X-ray crystallography. Improved sample quality achieved with stabilized receptors may also facilitate structural elucidation via alternative approaches, such as cryo-EM.

## Results

Here, we describe the YDDS (Yeast Direct Detergent Screening), a yeast-based directed-evolution approach, in which the selection of GPCR variants is facilitated by their ability to maintain the native fold and thus binding of a fluorescent ligand in the presence of short-chain detergents. In contrast to bacteria, the yeast’s protein synthesis, maturation, quality control (QC) and membrane trafficking machineries, including ER folding chaperones, and enzymes assisting disulfide bond formation and glycosylation, support the production of membrane receptors in a functional form^31,32^. More importantly, in yeast the presence of a detergent-resistant cell wall offers a unique compartmentalization opportunity to link the phenotype of receptor variants expressed (resistance to a detergent) with their encoding DNA, thus affording the identification of stable GPCR variants with unparalleled efficiency.

### Setup of screening system

**Figure 1:**
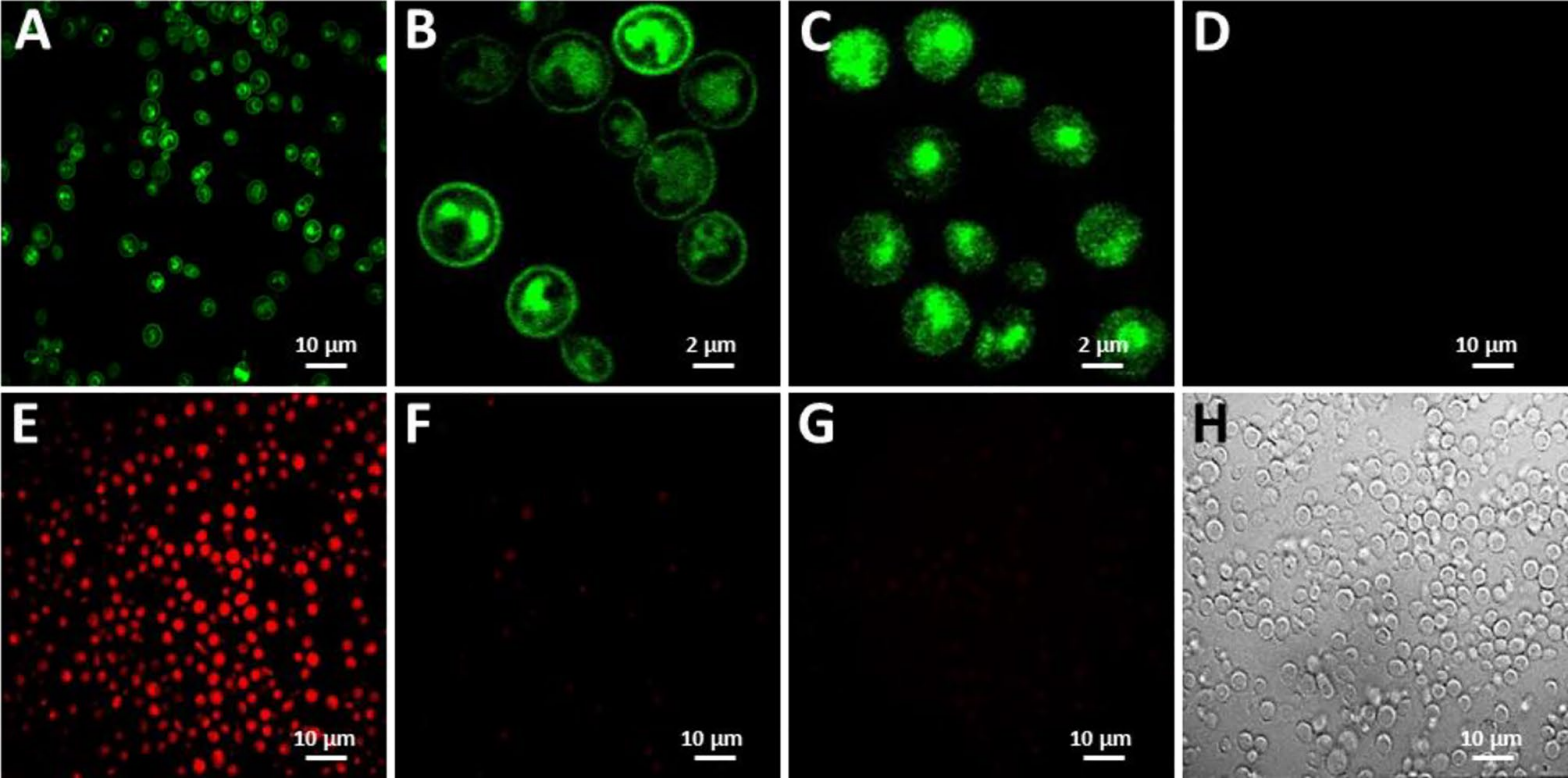
Detergent-resistant cell wall of yeast enables physical linkage between the phenotype and genotype of membrane-expressed A2aR. (**A**,**B**) Fluorescent confocal microscope images of yeast expressing WT-A2aR-eGFP (C-term) fusion. Some receptors appear to be trapped in ER. (**C**) A2aR-eGFP-expressing cells after 4 h incubation with 2% OG. The plasma membrane is dissolved and eGFP fluorescence is scattered throughout the cell volume. ER-retained receptors are visible. (**D**) Confocal microscope images of intact yeast expressing A2aR-WT (without eGFP) incubated for 1 h with 20 nM fluorescent A2aR agonist CA200623. Intact cells are impermeable to the ligand. (**E**) As in (**D**), only incubation with CA200623 was performed after yeast permeabilization by a brief (1 min) exposure to 1% OG. Membrane-expressed A2aR is properly folded and able to bind the fluorescent ligand. (**F**) Cells not expressing A2aR were treated as in (**E**). (**G**) As in (**E**), followed by 4 h incubation with 2% OG (in the presence of CA200623). The fluorescent signal is lost as a result of A2aR-WT denaturation. (**H**), Yeast cells treated with 2% OG for 4 h maintained their shape and mechanical integrity (white field).

As a model GPCR, we used human adenosine A2a receptor (A2aR), for which structurally stable variants have been developed by using conventional methods^19,20,33^. Thus, the YDDS could be compared to the existing methods in terms of the efficiency with which structure-stabilizing mutations are identified. Using A2aR-eGFP fusion, we and others^34–36^ demonstrated that A2aR is expressed and trafficked to the plasma membrane of *S. cerevisiae* yeast, although a significant fraction of the overexpressed receptor appears to retain in ER (Fig. 1A,B).

In the YDDS, the ability of the cell wall to maintain its mechanical integrity in the presence of a short-chain detergent, such as OG, is critical to confine the receptor—OG micelles and encoding plasmid DNA inside the cells. At the same time, free diffusion of a fluorescent ligand across the cell wall to reach membrane receptors is requisite. Using *S. cerevisiae* yeast expressing A2aR-WT, we demonstrated that the yeast cell wall is impermeable to molecules as small as CA200623 (925 Da), a fluorescent analog of adenosine receptor agonist NECA (Fig. 1D). However, cells that were exposed briefly (1 min) to 1% OG prior to CA200623 addition produced a strong fluorescent signal (Fig. 1E), which was absent in control cells, which did not express A2aR-WT (Fig. 1F). This indicates that the brief exposure to 1% OG was sufficient to perforate the cell wall, providing CA200623 with an access to membrane A2aR, while it was not harsh enough to denature A2aR-WT, which maintained its ability to bind the ligand. The integrity of the plasma membrane, however, appears to be compromised by the treatment, possibly resulting in membrane fragmentation, as could be inferred from the confocal microscope images demonstrating a homogeneous distribution of the fluorescent signal throughout the cell volume (not limited to plasma membrane) (Fig. 1E). The notion that cell membrane was irreversibly damaged by the brief exposure to 1% OG is consistent with the inability of the cells to recover after the treatment (Supplementary Fig. S1).

**Figure 2:**
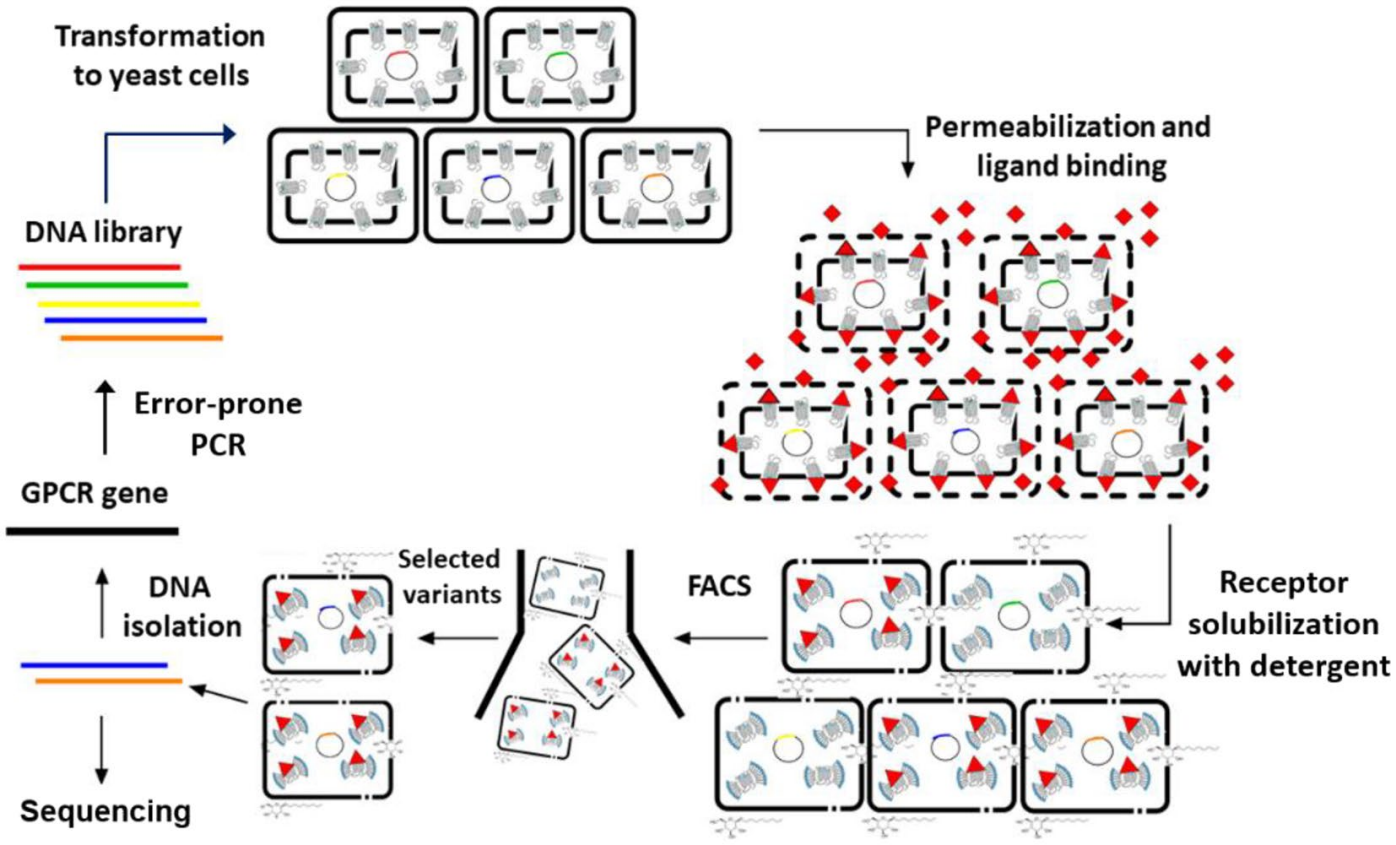
Workflow of YDDS. The evolution of a GPCR starts with a random mutagenesis of the receptor sequence using an error-prone PCR. A yeast library of GPCR variants is then generated by homologous recombination. GPCR-expressing cells are permeabilized and incubated with a fluorescent ligand (in red), followed by the treatment with a short-chain detergent (OG) to solubilize the plasma membrane and receptor. Subsequently, cells, which express receptor variants stable in the presence of OG are isolated by FACS and genotyped.

In contrast to the brief exposure to 1% OG, a prolonged (> 2 h) incubation with 2% OG resulted in a complete loss of the fluorescent signal in A2aR-WT expressing yeast (Fig. 1G), indicating membrane dissolution and the formation of A2aR-OG micelles, in which A2aR, as expected for the WT receptor, was denatured^20^. The dead cell carcasses thus formed, however, retained the overall shape and mechanical integrity of untreated cells (Fig. 1H, white field). The ability of the cell wall to maintain its mechanical properties in the presence of OG was also evident by the fluorescent confocal microscope imaging of yeast expressing A2aR-eGFP, in which the prolonged treatment with 2% OG resulted in a redistribution of eGFP fluorescent signal (resistant to OG treatment) from plasma membrane to cytosol (Fig. 1B,C), suggesting the formation of A2aR-eGFP—OG micelles. Both micelles and receptor-encoding plasmid DNA remained confined within the cell carcasses (accessed by sequencing of the plasmid DNA extracted from the cell carcasses). FACS could thus be used to isolate clones that express A2aR variants, whose overall fold and ligand-binding ability remained intact in the presence of short-chain detergents (Fig. 2). In our further experiments, we used A2aR without eGFP-tag, because A2aR-eGFP fusion failed, for as yet unknown reasons, to bind fluorescent A2aR ligand (not shown).

**Figure 3:**
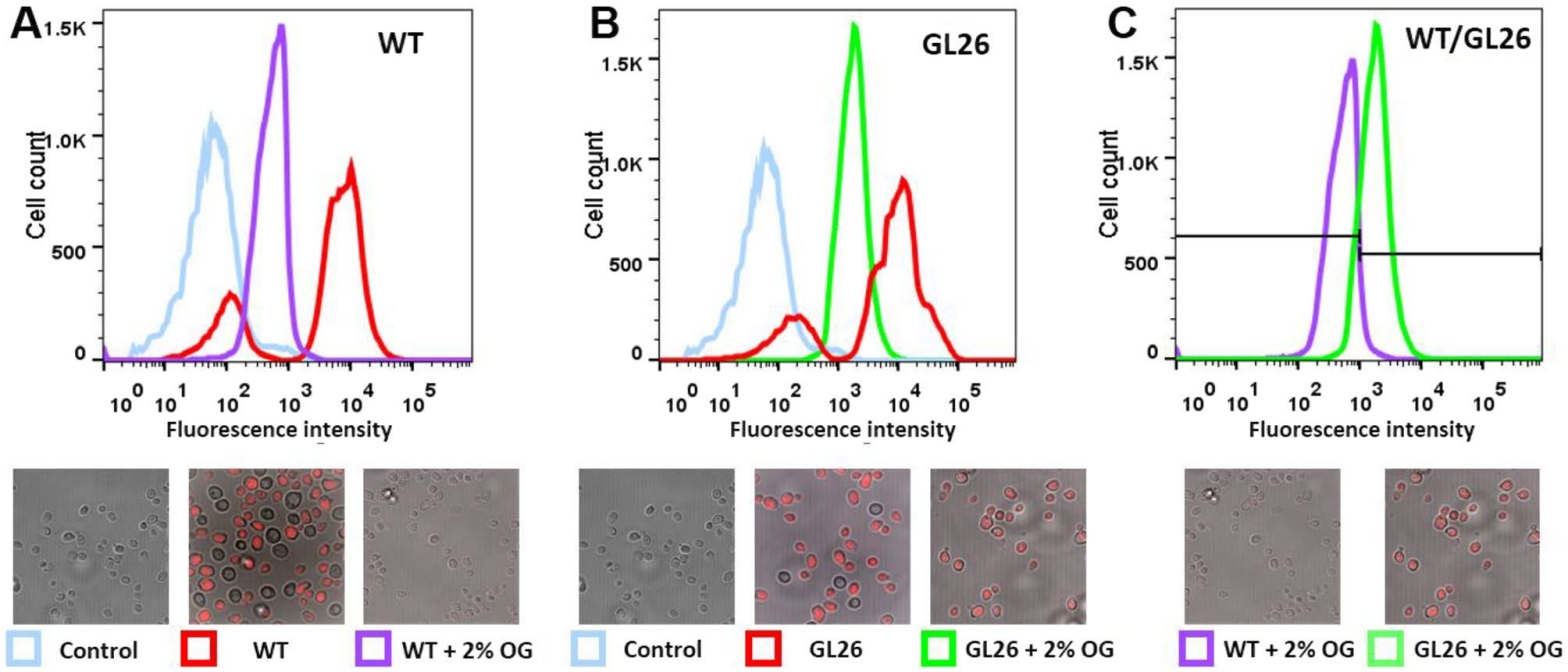
OG-resistant and unstable A2aR variants could be separated by flow cytometry of yeast. Permeabilized yeast expressing A2aR-WT (**A**) or thermostable GL26 mutant (**B**) were preincubated for 1 h with 20 nM CA200623, followed by 4 h incubation in the absence (red) or presence (purple or green) of 2% OG and CA200623, and after washing, analyzed by flow cytometry. Cells not expressing A2aR were used as control (light blue). (**C**) Fluorescence peaks corresponding to A2aR-WT (**A**) and GL26 (**B**) expressing cells treated with 2% OG are superimposed. Representative confocal microscope images of the samples analyzed by FACS are shown at the bottom.

To validate the system, we expressed a known thermostable A2aR variant, namely GL26, harboring a combination of four independent thermostabilizing mutations identified by an alanine-scan mutagenesis^19^. After permeabilization, the A2aR-WT and GL26-expressing cells were preincubated with 20 nM CA200623 for 1 h, followed by 4 h incubation in the presence of 2% OG (and 20 nM CA200623), and after washing, analyzed by flow cytometry. As shown in Fig. 3A,B, unlike A2aR-WT, GL26-expressing yeast retained a significant measure of fluorescence after OG treatment, resulting in an almost complete separation of A2aR-WT and GL26 cell populations on the basis of their residual fluorescence intensity (Fig. 3C). Thus, residual fluorescence could be used to separate yeast populations expressing OG-resistant and unstable (WT-like) A2aR variants by FACS.

### Library construction and screening

**Figure 4:**
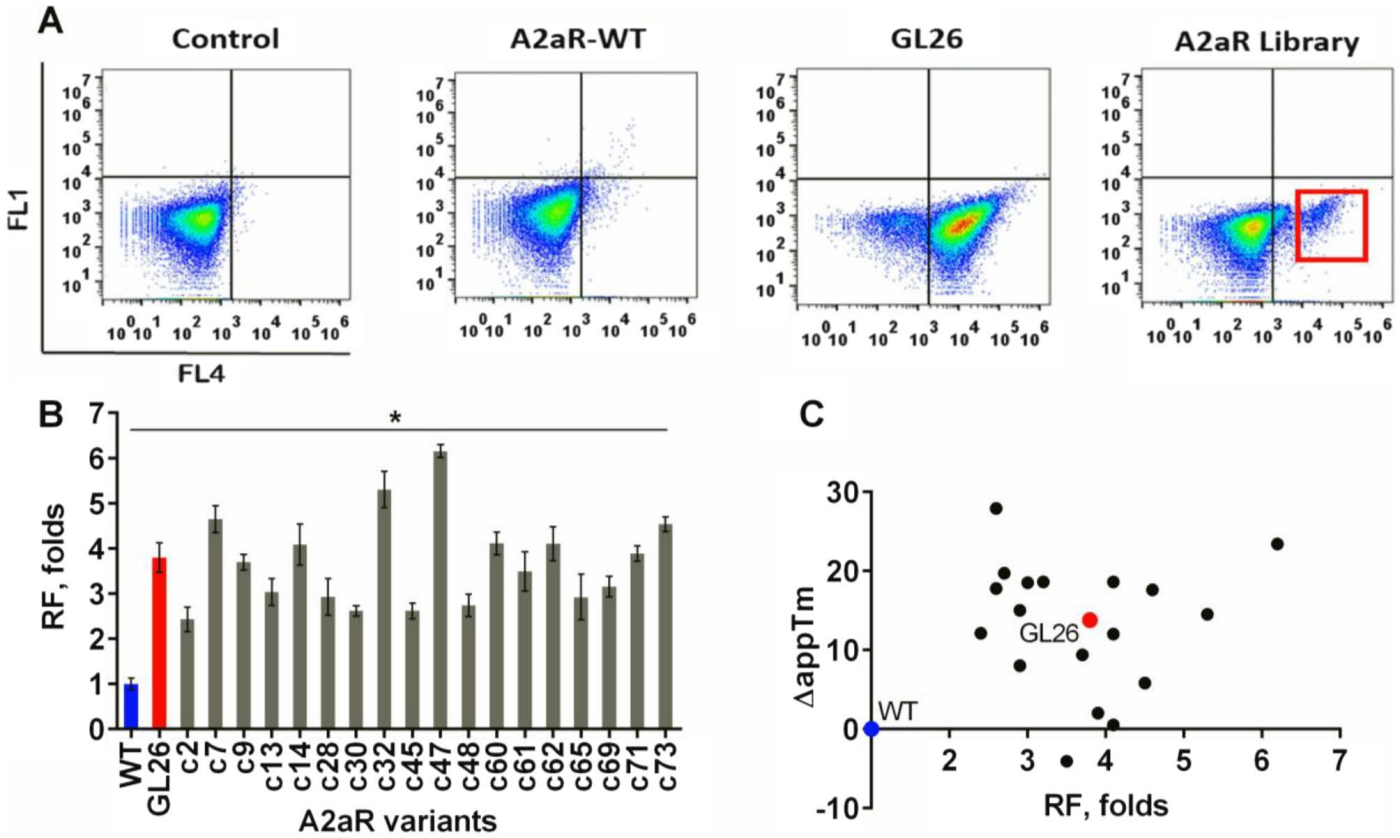
YDDS retrieves A2aR variants with various stability profiles. (**A**) Permeabilized yeast expressing A2aR-WT, GL26 mutant or library of A2aR mutants were preincubated with 20 nM CA200623 for 1 h, treated with 2% OG for 4 h (see “Methods”), and analyzed by FACS. FL4-APC channel was used to detect CA200623 fluorescence, while FL1-FITC channel (negative control) was added for the dot plot generation. Cells not expressing any receptor were used as control. The red square outlines the unique cell population present in the A2aR library, which remained fluorescent after OG treatment. (**B**) Encoding DNA isolated from the OG resistant cell population present in the A2aR library (red square in **(A)**) was reintroduced into yeast by a homology recombination, followed by the OG resistance analysis of randomly selected clones using flow cytometry, as described in (**A**). Eighteen clones with the highest residual florescence after OG treatment, RF (geometric MFI across the population, relative to that of A2aR-WT), were selected for further analyses. The RF values reported are the mean ± SE of three independent experiments performed in triplicate. An unpaired parametric t-test was used to evaluate the significance of the differences between A2aR-WT and OG resistant variants. Statically significant differences were found between all the groups compared, * p < 0.005. (**C**) The Pearson correlation analysis detects no correlation between the extent of thermostabilization (ΔappTm) determined by the whole-yeast thermostability assay and RF. The A2aR-WT and GL26-expressing yeast are indicated as blue and red dots, respectively. To construct the plot, the mean values of ΔappTm and RF of three independent experiments were used.

We next generated a library of A2aR variants (1 × 10^7^ clones), to harbor random mutations at a rate of 3–6 amino acid substitutions per clone, using an error-prone PCR and the complete receptor sequence as template. The mutation rate was chosen based on the results of the previous A2aR stabilization studies, indicating the requirement of a combination of multiple independent mutations to achieve a significant degree of structural stability^19,20^. The flow cytometry analysis of A2aR library-expressing yeast, permeabilized and treated—after CA200623 binding—with 2% OG (as described above), revealed the presence of a unique cell population, absent in A2aR-WT-expressing yeast, whose fluorescence profile matched that of GL26-expressing cells (Fig. 4A). Since yeast were not viable after OG treatment, the plasmid DNA isolated from the fluorescent population (~ 1% of the total number of cells sorted) was used as template for PCR-amplification of the receptor-encoding region, which was then reintroduced into yeast by a homology recombination to yield the YDDS library, a sub-library enriched in OG-resistant A2aR variants. The stability properties of A2aR variants expressed in seventy-five randomly selected clones from the YDDS library were analyzed by CA200623 binding in the presence of 2% OG (Supplementary Fig. S2). The encoding DNA isolated from the clones with the highest residual fluorescence (Fig. 4B) were sequenced, and the amino-acid sequence alignment is shown in Supplementary Fig. S3, with the mutation positions summarized in Supplementary Table S1.

### Functional characterization of A2aR variants resistant to OG

**Figure 5:**
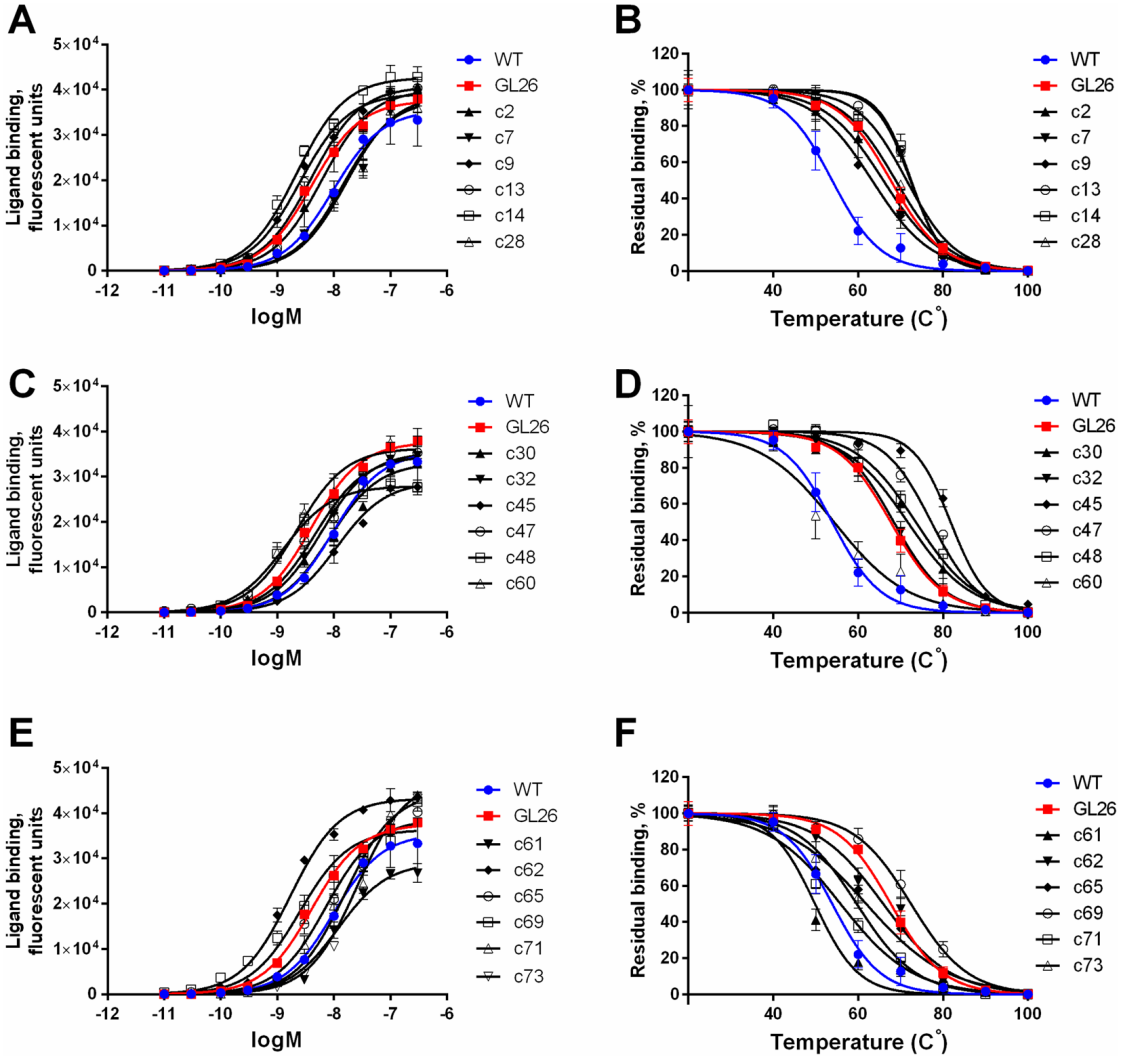
Expression, affinity to agonist and thermostability of OG resistant A2aR variants retrieved by YDDS. (**A,C,E**) Permeabilized yeast expressing OG resistant A2aR variants were incubated for 1 h with the indicated concentrations of CA200623, and after washing, analyzed by flow cytometry. A three-parameter logistic regression model^32^ was used to analyze the experimental data. (**B,D,F**) Yeast expressing OG resistant A2aR variants were heated for 30 min at the indicated temperatures, cooled, permeabilized and incubated for 1 h with 20 nM CA200623 in the presence of 1% DM, and after washing, analyzed by flow cytometry. Data were analyzed by a non-liner regression using Boltzmann sigmoidal function. Data from one representative experiment of three independent experiments is presented as the mean ± SD of three technical replicates.

A saturation ligand binding assay was performed to assess the effect of the structure-stabilizing mutations revealed by the YDDS on the level of A2aR membrane expression and affinity to CA200623 agonist. The analysis of the OG resistant variants, including GL26, (Fig. 4B) revealed that their apparent affinity to CA200623 agonist was similar to that of A2aR-WT (Fig. 5A,C,E and Table 1). Similar results were reported by Lebon et al*.*, indicating that mutations stabilizing *E. coli*-expressed A2aR in an agonist (NECA)-bound conformation failed to increase significantly the receptor's affinity to agonists^19^, as it would be expected for the receptor stabilized in a fully activated conformational state^37,38^. These observations may reflect the paradigm, according to which the formation of the agonist-receptor-G protein ternary complex is required for the receptor to assume a fully activated state^39^. The lack of G protein complement for GPCRs expressed in either prokaryote or yeast may be accountable for the inability of the screening methods to identify receptor variants stabilized in the fully activated state (rather than in an intermediate state along the activation pathway). GPCR co-expression with a corresponding G protein may constitute a strategy to overcome such limitation.

**Table 1:** Functional characterization of OG resistant A2aR variants retrieved by YDDS.

A2aR variant	Expression, B_max_ ± SE^a^	Expression, fold^b^	logEC_50_ ± SE^c^	appT_m_ ± SE^d^	ΔappT_m_^e^	RF ± SE, fold^f^
WT	35,541 ± 1111	1.00	−8.01 ± 0.06	53.9 ± 0.6	0.0	1.0 ± 0.07
GL26	37,625 ± 664	1.06	−8.39 ± 0.04	67.7 ± 0.4	13.8	3.8 ± 0.19
c2	40,003 ± 1289	1.13	−8.21 ± 0.07	66.0 ± 0.8	12.1	2.4 ± 0.16
c7	38,859 ± 1001	1.09	−7.78 ± 0.04	71.5 ± 0.7	17.6	4.6 ± 0.17
c9	39,208 ± 655	1.10	−8.60 ± 0.04	63.3 ± 0.3	9.4	3.7 ± 0.10
c13	40,703 ± 707	1.15	−8.40 ± 0.04	72.4 ± 0.2	18.5	3.0 ± 0.17
c14	42,728 ± 651	1.20	−8.66 ± 0.03	72.5 ± 0.5	18.6	4.1 ± 0.26
c28	37,924 ± 1161	1.07	−7.84 ± 0.05	68.9 ± 0.6	15.0	2.9 ± 0.23
c30	33,098 ± 818	0.93	−8.07 ± 0.05	71.7 ± 0.6	17.8	2.6 ± 0.07
c32	35,403 ± 520	1.00	−8.22 ± 0.03	68.4 ± 0.4	14.5	5.3 ± 0.23
c45	28,931 ± 654	0.81	−7.95 ± 0.04	81.8 ± 0.4	27.9	2.6 ± 0.10
c47	34,607 ± 898	0.97	−8.33 ± 0.05	77.3 ± 0.5	23.4	6.2 ± 0.08
c48	27,918 ± 379	0.79	−8.90 ± 0.03	73.6 ± 0.7	19.7	2.7 ± 0.14
c60	36,322 ± 815	1.02	−8.65 ± 0.05	54.4 ± 1.2	0.5	4.1 ± 0.15
c61	29,034 ± 702	0.82	−7.93 ± 0.04	49.8 ± 0.8	-4.1	3.5 ± 0.25
c62	43,305 ± 652	1.22	−8.77 ± 0.04	65.9 ± 0.9	12.0	4.1 ± 0.22
c65	38,688 ± 1038	1.09	−8.14 ± 0.05	61.9 ± 1.0	8.0	2.9 ± 0.29
c69	36,390 ± 1183	1.02	−8.60 ± 0.07	72.5 ± 0.5	18.6	3.2 ± 0.13
c71	44,651 ± 1482	1.26	−7.80 ± 0.06	55.9 ± 0.6	2.0	3.9 ± 0.09
c73	47,582 ± 956	1.34	−7.59 ± 0.03	59.7 ± 0.6	5.8	4.5 ± 0.09

^a^The level of receptor expression was estimated by ligand binding at saturating concentrations of CA200623 (B_max_). The B_max_ ± SE values were calculated from the data shown in Fig. 5A,C,E by a nonlinear regression, as described in the Methods.

^b^The level of receptor expression relative to that of A2aR-WT, folds.

^c^As a measure of apparent affinity we used ligand concentration at the receptors' fractional occupancy of 0.5 (EC_50_), estimated from the sigmoid concentration–response curves (Fig. 5A,C,E) using a three-parameter logistic regression model^32^. LogEC_50_ were reported as the mean ± SE of three independent experiments performed in triplicate.

^d^Apparent T_m_ (appT_m_) determined by the whole-yeast thermostability assay was defined as a temperature at which CA200623 binding measured at 22 ºC is reduced by 50%, and was reported as the mean ± SE of three independent experiments performed in triplicate.

^e^Receptor thermostabilization (ΔappT_m_) was defined as appT_m_ (A2aR variant) − appT_m_ (A2aR-WT).

^f^RF was defined as a residual fluorescence measured in cells incubated with 20 nM CA200623 and treated with 2% OG. The values are relative to RF of A2aR-WT, and are expressed as the mean ± SE of three independent experiments performed in triplicate.

Another notable observation was that OG resistance did not result in an increased plasma membrane expression of A2aR variants. As a measure of receptor expression we used the fluorescence signal obtained at saturating concentrations of CA200623 (Fig. 5A,C,E and Table 1). The result suggests that correlation between the level of membrane expression and structural stability is not a universal phenomenon for GPCRs. It is possible that A2aR-WT transport to the plasma membrane already operates at its maximal capacity, a result of the saturation of certain element(s) of the transport machinery, which are different from the QC system of ER, whose role is to prevent structurally unstable species from reaching the membrane^40^. Thus, membrane expression of A2aR in the given system could not be further improved by increased structural stability of the receptor. The results are consistent with the findings by Magnani et al*.*, demonstrating the lack of correlation between thermostability of A2aR variants and the level of their membrane expression in *E. coli*^20^. Combined, the results stress the notion that expression-guided selection might not be a universal strategy for GPCR stability screening, and properties that are directly related to structural stability should be used instead.

Having said that, however, a certain precaution should be used in interpreting the results of the ligand binding experiments employing yeast cells permeabilized as described above. The permeabilization by a brief (1 min) exposure to 1% OG appears to impair the integrity of the plasma membrane (Fig. 1E), raising the possibility that ER membrane may also be affected by the treatment. In this case, ER-retained receptors, whose ligand-binding sites face ER lumen, may participate in ligand binding, resulting in an inaccurate quantitation of plasma membrane receptors. We, however, consider such possibility unlikely. As seen in Fig. 1B,C, the harsh treatment with 2% OG for 4 h did nor scatter the clusters of A2aR-eGFP fusion receptors retained in ER, indicating that the treatment did not fragment ER membrane. We, therefore, assume that a much milder treatment with 1% OG for 1 min, which we used for yeast wall permeabilization, does not compromise the integrity of ER membrane.

Thermostability is a parameter conveniently employed to envisage GPCR behavior in the presence of harsh detergents^19,20^. The mechanism of protein denaturation inside detergent micelles, however, is poorly understood^18,41^. Even thermal and chemical (by urea or guanidinium chloride) denaturations, considered related^42^, exhibit distinct thermodynamic profiles^43^, emphasizing the notion that different methods of denaturation should not be treated as similar as a matter of convenience, but instead, considered within a framework of effects, such as electrostatic and solvophobic, which dominate protein interactions with its immediate environment^43^. Here, we used A2aR variants retrieved by the YDDS to analyze correlation between receptor stability in the presence of short-chain detergents and thermostability.

Thermostability is usually assessed in receptors solubilized by a mild detergent, such as DM (*n*-decyl-β-d-maltopyranoside) or DDM (*n*-dodecyl-β-d-maltoside), and optionally purified by chromatography^19,44,45^. To simplify the analysis, we employed a whole-yeast thermostability assay, which does not require membrane solubilization and receptor isolation/purification. In the method, intact A2aR-expressing yeast are heated to a desired temperature for 30 min, cooled and permeabilized by a brief exposure to 1% OG. The permeabilized yeast are then incubated at 22 ºC for 1 h with 20 nM CA200623 in the presence of 1% DM, and after washing, the residual fluorescence is evaluated by flow cytometry. For A2aR-WT, the apparent T_m_ (appT_m_), a temperature at which ligand binding measured at 22 ºC is reduced by 50%, determined by the whole-yeast thermostability assay was 53.9 ºC, which is ~ 31 ºC higher than that measured by the conventional method (23 ºC)^20^ (Fig. 5B and Table 1). Such difference may stem, at least partially, from the stabilizing effect of the native environment of lipid bilayer during heating. The gain in thermostability (ΔappT_m_) for GL26 mutant relative to A2aR-WT determined by the whole-yeast assay was 13.8 ºC, as compared to 21.5 ºC measured by the conventional method^19,20^ (Fig. 5B and Table 1). For all OG resistant A2aR variants (Fig. 4B), the appT_m_ was determined using the whole-yeast assay (Fig. 5B,D,F) and calculated ΔappT_m_ values were compared to the extent of the variants' resistance to OG. Since all the OG resistant A2aR variants exhibited similar levels of membrane expression and affinity to CA200623 (Fig. 5A,C,E and Table 1), as a measure of OG resistance we used the residual fluorescence (RF) of the cells incubated with 20 nM CA200623 and treated with 2% OG (Fig. 4B). No significant correlation was detected between the level of A2aR variant thermostabilization (ΔappT_m_) and OG resistance, Fig. 4C. For instance, highly thermostable variant c45 was only moderately resistant to OG (Figs. 4B, 5D and Table 1), while thermostability of fairly OG resistant c61 variant was even lower than that of A2aR-WT (Figs. 4B, 5F and Table 1). These observations support the notion that different methods of evaluating structural stability are not equivalent, stressing the importance of using screening strategies, in which clone selection is carried out directly based on the desired property, which in our case was stability in the presence of short-chain detergents required for vapor diffusion crystallography. This would increase the probability of identifying high-quality receptor variants, and reduce the costs and time associated with the development of candidates suitable for crystallization and structural elucidation.

## Discussion

Here, we presented the YDDS, a yeast-based screening platform for a single-step isolation of GRCR variants stable in the presence of short-chain detergents. The unique property of yeast—a detergent resistant cell wall—physically links the receptor's phenotype with its encoding genetic material. Combined with the advantages of the eukaryotic protein synthesis and transport machineries, the methodology offers a fast, and efficient alternative to the existing approaches of GPCR structural stabilization, most of which employing a selection criterion related to the receptor's property of interest (crystallizability) only circumstantially.

**Figure 6:**
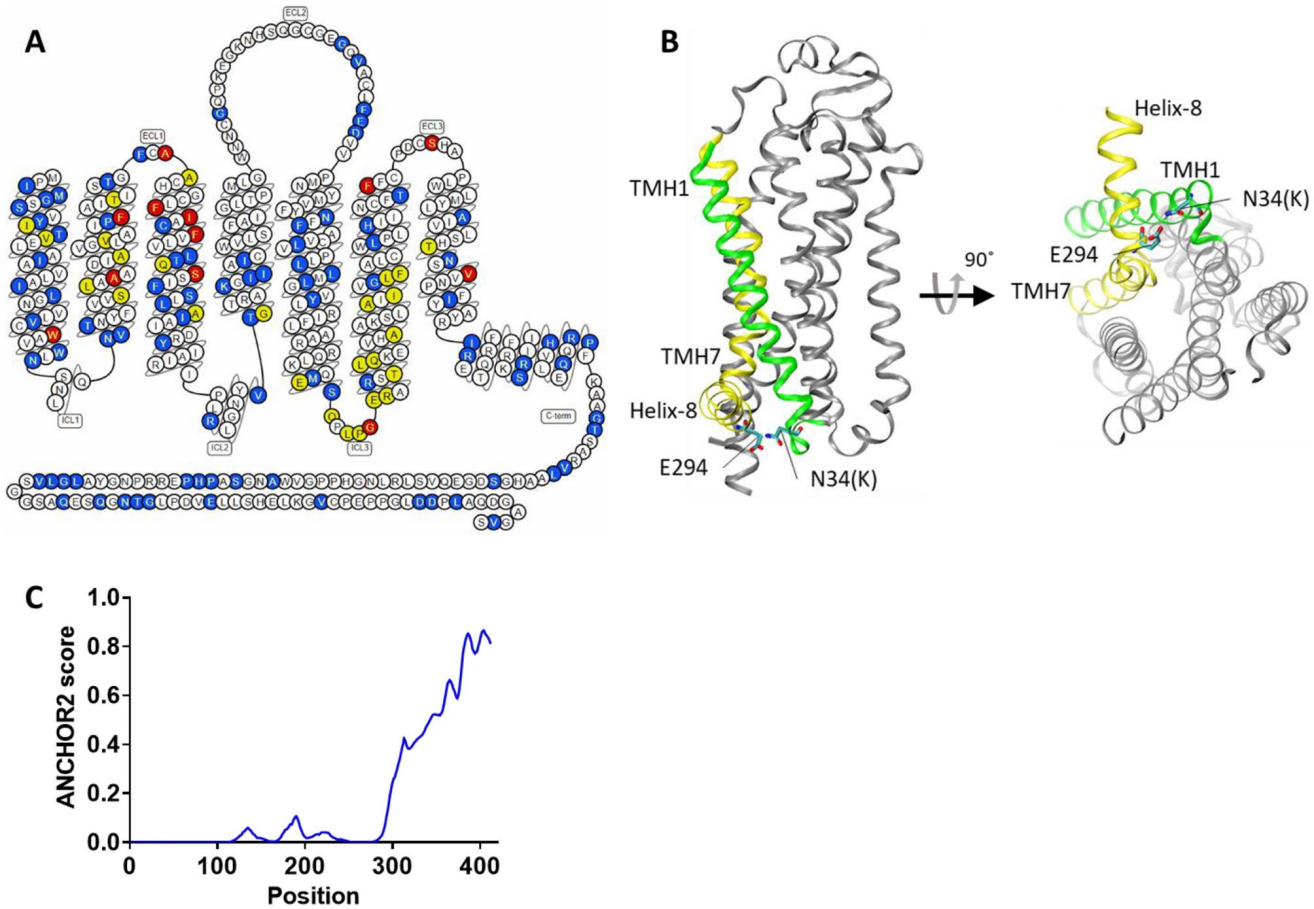
A2aR scaffold is highly amenable to stabilization. (**A**) The snake-plot of A2aR-WT highlights the positions of stabilizing mutations. In yellow—positions of Ala/Leu substitutions conferring thermostability upon the agonist-bound receptor^12^. In blue—positions of substitutions occurring in OG resistant A2aR variants identified by the YDDS (this study). In red, position identified by both studies. (**B**) A single N34K substitution in c7 variant confers OG resistance and thermostability upon A2aR. The A2aR structure (PDB: 4UG2) is shown in cartoon representation. The TMH1 and TMH7 are colored in green and yellow, respectively. (**C**) The C-terminus of A2aR is intrinsically disordered. The ANCHOR2 plot shows the probability (indicated by ANCHOR2 score) of the given A2aR-WT residue being part of a disordered binding region (default settings were used)^34^.

The repertoire of A2aR mutations revealed by the YDDS in association with OG resistance provides important insights into the structural basis of GPCR stability. In contrast to the previous studies focused on A2aR thermostability^19,20^, we used the resistance to short-chain detergents—a prerequisite for successful vapor diffusion crystallization—as a primary criterion for clone selection. Having said that, out of 38 previously reported positions, where Ala/Leu substitutions improved thermostability of agonist-bound A2aR, 12 were recaptured by the YDDS, including 6 out of 16 positions with the highest stabilizing effect^19,20^ (Fig. 6A, Supplementary Fig. S3 and Supplementary Table S1). Not all the previously described positions were retrieved in the present study, which could be explained by the actual screening of only a fraction of the original A2aR library (by FACS), and also by an insufficient sampling of the enriched YDDS library for sequencing (only seventy-five randomly selected clones were sequenced). Combining the YDDS with a massive parallel sequencing would enable the entire sequence space associated with receptor stability in the presence of short-chain detergents to be revealed. In addition, the YDDS uses a different criterion for clone selection (OG resistance) compared to that of the previous work (thermostability)^19,20^, which—as we showed above (Fig. 4C)—are not equivalent.

Although caution should be exercised in interpreting the YDDS mutagenesis data, as not all the mutations occurring in the OG resistant clones might indeed contribute to the desired phenotype (a comprehensive reverse mutagenesis study is required to assess the contribution of individual mutations and their various combinations to OG resistance), the YDDS identified a multitude of A2aR positions which were not described previously. A large number of positions with a potential structure-stabilizing effect suggests that the GPCR scaffold might not be intrinsically unstable, but had rather evolved to accommodate a great measure of conformational flexibility and structural dynamics essential for receptor functioning. In particular, conformational dynamics is essential for GPCR activity, the process in which the receptor assumes a conformation(s) capable of coupling to and activating the intracellular effector protein(s)^46^. Structural instability might be incorporated via the so-called stability "cold spots", residues whose side-chains interfere with tight packing of local structures. The removal of such cold spots, e.g., by substitution to alanine, may resolve sterical clashes, improve local packing, and increase stability of the protein as whole. Consistent with this view is the notion that the enthalpic component resulting from the decreased intra-molecular tension and improved inter-residue contacts appears to contribute a significant portion of the free energy of stabilization in the thermostable GPCR mutants^47–49^. The existence of the cold spots is evidenced by the fact that alanine-scan mutagenesis was capable of conferring structural stabilization upon GPCRs^19,20^. Moreover, local structural perturbations introduced by the cold spot(s), and their destabilizing effect on the global protein structure, might be counterbalanced (at least partially) by a limited number of stabilizing mutations placed at various influential positions throughout the GPCR scaffold. The YDDS method is capable of exposing such positions, since, in contrast to Ala scan, which mostly removes sterical clashes, it operates by introducing novel functionalities into the existing molecular background, thus more completely utilizing the existing or creating new interaction potential. A remarkable example of such compensatory mutations is provided by variant c7 retrieved by the YDDS, in which a single N34K substitution renders A2aR resistant to OG, and also confers it a substantial measure of thermostability exceeding that of GL26 mutant (containing a combination of four independent Ala/Leu substitutions^19^) (Table 1, Supplementary Fig. S3 and Supplementary Table S1). The N34 residue is located in the intracellular end of the transmembrane helix 1 (TM1), and it is H-bound to E294 in the intracellular end of the TM7, Fig. 6B. The TM1 and TM7, the most remote helices in the primary sequence, come to a close proximity in the barrel-like structure of the folded receptor's transmembrane domain (TMD), with their interface contacts contributing to the stability of the entire fold (Fig. 6B). This idea is consistent with the large number of structure-stabilizing mutations identified by the YDDS in the TM1 of A2aR (Fig. 6A and Supplementary Fig. S3 and Supplementary Table S1). The N34K substitution replaces the H-bond between N34 and E294 with a much stronger salt-bridge, further stabilizing the TMD fold (Fig. 6B). This example demonstrates that a single but strategically placed substitution may overcome the excessive conformational entropy of the GPCR scaffold, rendering it thermostable and resistant to short-chain detergents.

The vast majority of GPCRs have been crystalized with their C-terminus truncated. In addition to improved expression, C-terminus removal increases structural stability and homogeneity of receptor preparations, therefore improving crystal quality^50–52^. The deletion of the C-terminus, however, diminishes hydrophilic surfaces, commonly observed to form lattice contacts in membrane protein crystals, and could be considered counterproductive for crystallization efforts^53^. However, a large portion of the GPCR C-terminus (> 60% of residues on average throughout the family) has been shown to be intrinsically disordered^54^. This lack of ordered structure explains the beneficial effects of the C-terminus removal on receptor crystallizability. The intrinsically disordered regions (IDR) are known to undergo context-dependent transition between the unstructured and structured states, triggered by extrinsic factors such as interactions with folded proteins, alteration in local charge distribution due to pH changes, redox state, and PTM^46,54–57^. The GPCR C-tail has been proposed to utilize such induced-folding mechanism to mediate specific interactions with a multitude (more than 40) of cytosolic effector proteins, including promotion of oligomerization^58,59^. Over 400 PTM sites were reported to occur in the C-terminus region of human GPCRs, including phosphorylation, ubiquitination, methylation and acetylation; these PTM may function as interaction switches within the IDR, coordinating cellular responses and contributing to cell decision-making^54,60–63^. A particularly long C-terminus of A2aR (> 120 residues) was shown to act as docking site for kinases, β-arrestin, α-actinin, ARNO, USP4, translin-associated protein-X, and also contribute to the formation of heteromeric complexes with D_2_-dopamine receptor and the metabotropic glutamate receptor-5^64^. Our analysis of the A2aR structure using ANCHOR2 program^65^, predicted that the A2aR C-terminus (aa. No. > 300) is, with a high probability, an IDR expected to undergo a disorder-to-order transition triggered by exogenous factors (Fig. 6C). We therefore hypothesized that mutations in the C-terminus may induce, alike PTM, tail transition into a folded state^61–63,66–68^, which in turn may improve the stability properties of the entire receptor. From 18 OG resistant variants analyzed (Fig. 4B), 14 contained mutations in the C-terminus, spanning the entire putative IDR from position 304 (C-term part of the cytoplasmic Helix 8) to 412 (Fig. 6A,C and Supplementary Fig. S3 and Supplementary Table S1). The replacement or, instead, introduction of proline or glycine residues, a sequence alteration expected to affect local structure profoundly, accounted for one-third of all the substitutions occurring in the A2aR C-terminus. The impact of the C-terminus conformational state on the stability properties of the entire receptor was demonstrated in variants c48 and c73, each of which harbors only two substitutions (both substitutions in the C-terminus), sufficient to confer OG resistance upon A2aR. The substitution of two proline residues (P313S and P354A) accounted for OG resistance and marked thermostability of c48 variant (Table 1 and Supplementary Fig. S3 and Supplementary Table S1). Whereas it remains to be tested whether C-terminus-mediated structural stabilization is sufficient to facilitate GPCR crystallization, the paradigm may offer a practical alternative to commonly exercised strategies of mounting hydrophilic surfaces and decreasing excessive flexibility by fusing the receptor with a well-folded auxiliary protein, or by interacting it with a specific nano- or antibody^50^.

The prerequisite for the YDDS is the availability of a fluorescent ligand, either small-molecule or peptide capable of equilibrating across the perforated cell wall of yeast. Such ligands are currently available for a wide variety of GPCRs^69–74^, and the list is constantly growing. The proposed methodology is not limited to GPCRs and could be employed to improve structural stability of other membrane proteins, such as ion channels, which are also important drug targets and difficult to crystallize.

## Methods

### A2aR expression in yeast

The pITy-MC-His_10_ and pITy-MCeGFP-His_10_ expression vectors encoding for A2aR-WT and A2aR-WT-eGFP, respectively, were a kind gift of Dr. Anne S. Robinson (University of Delaware, Newark, USA). These vectors contained a synthetic pre-pro leader sequence (KVLIVLLAIFAALPLALAQPVISTTVGSAAEGSLDKR) preceding the receptor sequence, whose addition was shown to be critical for efficient A2aR expression in yeast^34^. The expression was driven by Gal1-10 promotor. The plasmids were transformed into *S. cerevisiae* yeast EBY100 strain using electroporation as described^75^. After transformation, cells were grown overnight in 5 ml YPD media [2% peptone (BD Becton Dikinson, USA), 2% glucose (Sigma-Aldrich, Israel), 1% yeast extract (Gibco, USA)] at 30 °C (290 rpm), and A2aR expression was induced by transferring the cells (at OD_600_ = 0.5) to YPG induction media [2% peptone, 2% galactose (Sigma-Aldrich, Israel), 1% yeast extract]. For screening experiments, A2aR-WT encoding sequence (including the N-terminal pre-pro leader sequence) was subcloned into a high-copy episomal pYES2 vector (encoding for *URA3* gene) for galactose-inducible protein expression (a kind gift of Dr. Maya Schuldiner, Weizmann Institute of Science, Israel). To afford the use of URA3 marker for transformant selection, *S. cerevisiae* BY4741 strain (*MAT*α *his3Δ1 leu2Δ0 met15Δ0 ura3Δ0) *^76^ was selected for further experiments.

The yeast A2aR library was created using a homologous recombination by transforming BY4741 yeast with a linearized pYES2 vector and the product of A2aR error-prone PCR (see *A2aR library construction*), as described^75,77^. The cells were grown at 30 °C overnight in 5 ml synthetic complete (SC) medium containing 4% glucose (290 rpm), and protein expression was induced by transferring cells (at OD_600_ = 0.5) to SC medium containing 4% galactose.

### Confocal microscopy analyses

Yeast expressing A2aR-eGFP fusion were grown to OD_600_ = 1.0, centrifuged at 2,500 × g for 3 min, and after washing imaged using Inverted Axio Observer Z1 Confocal Laser Scanning Microscope (Zeiss, Germany) and 40 × Water, 1.2 NA, DIC objective (Ex: 488 nm and Em: 511 nm). In experiments involving binding of CA200623 fluorescent ligand to A2aR-expressing yeast, Ex: 638 nm and Em: 657 nm filters were used.

### Ligand binding and OG resistance assays

The 200 μl of BY4741 yeast grown to OD_600_ = 1.0 were pelleted by centrifugation at 2500×*g* for 3 min, and after washing permeabilized by incubation at 22 °C for 1 min in phosphate buffered saline (PBS) in the presence of 1% OG (*n*-Octyl-β-d-glucopyranoside, Carl Roth, Germany) with shaking (600 rpm), followed by two washing steps with PBS. The permeabilized yeast were incubated at 22 °C for 1 h in PBS (1% DMSO) in the presence of the indicated concentrations of CA200623 (Abcam, UK), a fluorescent analog of nonspecific adenosine receptor agonist NECA, with shaking. After incubation, the cells were washed three times with PBS (1% DMSO), and incubated at 22 °C for additional 1 h in PBS (1% DMSO) (with shaking) to enable efficient removal of unbound ligand from the cells. After three additional washing steps with PBS, the cells were analyzed by flow cytometry using BD FACSCanto™ II Flow Cytometry System (BD Biosciences, USA) and APC (allophycocyanin) fluorescent channel (Ex: 638 nm, Em: 657 nm) for CA200623 detection. Each experimental condition was analyzed in triplicate. Geometric mean fluorescence intensity (MFI) was calculated using FlowJo software (Tree Star Inc., USA). Nonspecific binding was determined at each ligand concentration using A2aR non-expressing cells and the values were subtracted from the total binding. Saturation ligand binding data were analyzed by a non-linear regression using a three-parameter logistic function implemented in Prism6 software (GraphPad, USA).

To determine the extent of OG resistance of A2aR variants, BY4741 yeast were permeabilized as described above and incubated at 22 °C for 1 h in PBS (1% DMSO) in the presence of 20 nM CA200623. The cells were then incubated for additional 4 h at 22 °C in the presence of 2% OG and 20 nM CA200623 with shaking (600 rpm). After three washings steps + 1 h incubation with PBS (1% DMSO), total and non-specific binding were analyzed by flow cytometry, as described.

### A2aR library construction and sorting by flow cytometry

The A2aR library (1 × 10^7^ clones) carrying 3–6 random amino acid substitutions per sequence was generated by Gene Universal (USA), using error-prone PCR^78^ and the entire A2aR-WT sequence as template. The library was cloned into pYES2 vector and expressed in BY4741 yeast. After permeabilization, the cells were preincubated at 22 °C for 1 h with 20 nM of CA200623, followed by 4 h incubation with 2% OG in the presence of 20 nM CA200623, as described above. After washing, the cells were sorted by FACSAria™ III cell sorter (BD, USA), using APC channel (Ex: 638 nm, Em: 657 nm) for CA200623 detection and a neutral FITC channel (Ex: 488 nm, Em: 511 nm) for dot plot construction.

### Functional characterization of OG resistant clones

The sorted cells (dead cell carcasses) from the previous step (about 1% of the total number of cells analyzed by FACS) (~ 500 μl) were centrifuged at 2500×*g* for 3 min, and after washing solubilized by boiling in 10 μl NaOH (0.02 M) for 15 min, and after centrifugation, 2 μl supernatant was used to amplify the receptor-encoding sequence by PCR. The PCR product and a linearized pYES2 vector were transformed into BY4741 yeast to generate a sub-library enriched in OG resistant A2aR variants (the YDDS library) by homologous recombination. Structural stability of A2aR variants expressed in 75 clones randomly selected from the YDDS library were verified by CA200623 (20 nM) binding assay in the presence of 2% OG using flow cytometry, as described above. The encoding DNA of A2aR variants, whose OG stability was thus confirmed, were amplified in bacteria and submitted to Sanger sequencing.

For the whole-yeast thermostability assay, BY4741 yeast expressing A2aR-WT or OG resistant variants were incubated for 30 min at the indicated temperatures (22–100 °C) in PBS, followed by 30 min incubation at 22 ºC. The cells were then permeabilized with 1% OG, and incubated at 22 ºC for 1 h in PBS (1% DMSO) in the presence of 20 nM CA200623 and 1% *n*-decyl-β-d-maltopyranoside (DM), with shaking (600 rpm). After repetitive washings steps including 1 h incubation with PBS (1% DMSO), the cells were analyzed by flow cytometry, as described above. Nonspecific ligand binding was measured by using A2aR non-expressing cells and subtracted from the total binding. Data were analyzed by a non-liner regression using Boltzmann sigmoidal function implemented in Prism6 software.

### Statistical analyses

Statistical analyses of the presented data were performed by using Prism6 software (GraphPad, USA).

## Supplementary Information

Supplementary Information.

## Data Availability

The datasets generated during and/or analyzed during the current study are available from the corresponding author on reasonable request.
