# Supplementary material for: Yeast-based directed-evolution for high-throughput structural stabilization of G protein-coupled receptors (GPCRs)
Source: Sci Rep. 2022 May 23;12:8657. doi: 10.1038/s41598-022-12731-2 (PMC9126886; doi:10.1038/s41598-022-12731-2)
Supplement: Supplementary file 1 — Supplementary Information. [file 41598_2022_12731_MOESM1_ESM.pdf]

Supplementary information

Yeast-based directed-evolution for high-throughput structural stabilization of G protein-coupled receptors (GPCRs)

M. Meltzer, T. Zvagelsky, U. Hadad, N. Papo and S. Engel

Supplementary Fig. S1. The permeabilization treatment irreversibly damages yeast cells. The BY4741 yeast were treated with or without (control) 1% OG for 1 min as described in the Methods, and allowed to recover by incubation in a growth media at staring OD_{600} = 0.1 at 30 °C (275 rpm). The cells failed to recover after the permeabilization, indicating irreversible damage.

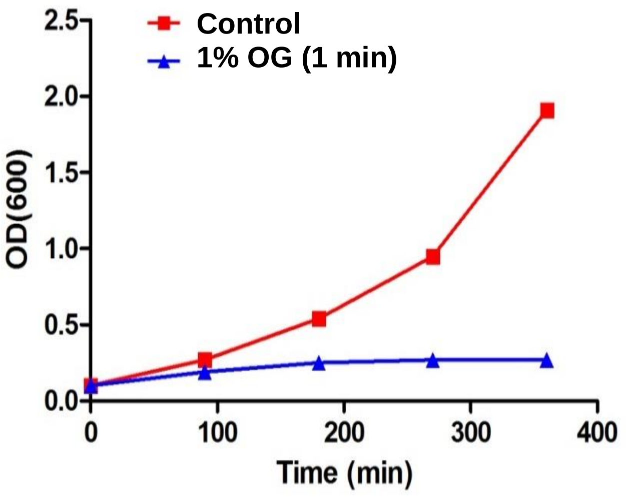

Supplementary Fig. S2. OG resistance profile of clones randomly selected from YDDS library. Encoding DNA isolated by PCR from the OG-resistant cell population present in the A2aR library was reintroduced into yeast by homology recombination to yield a sub-library enriched in OG-resistant A2aR variants (the YDDS library). Seventy five randomly selected clones from the YDDS library were analyzed using CA200623 (20 nM) binding assay in the presence of 2% OG, and the extent of residual florescence was measured by flow cytometry (as described in the Methods). Eighteen clones with the highest residual florescence (geometric MFI across the population) were selected for further analyses (colored orange). The bars representing A2aR-WT and thermostable mutant GL26 are colored in blue and green, respectively.

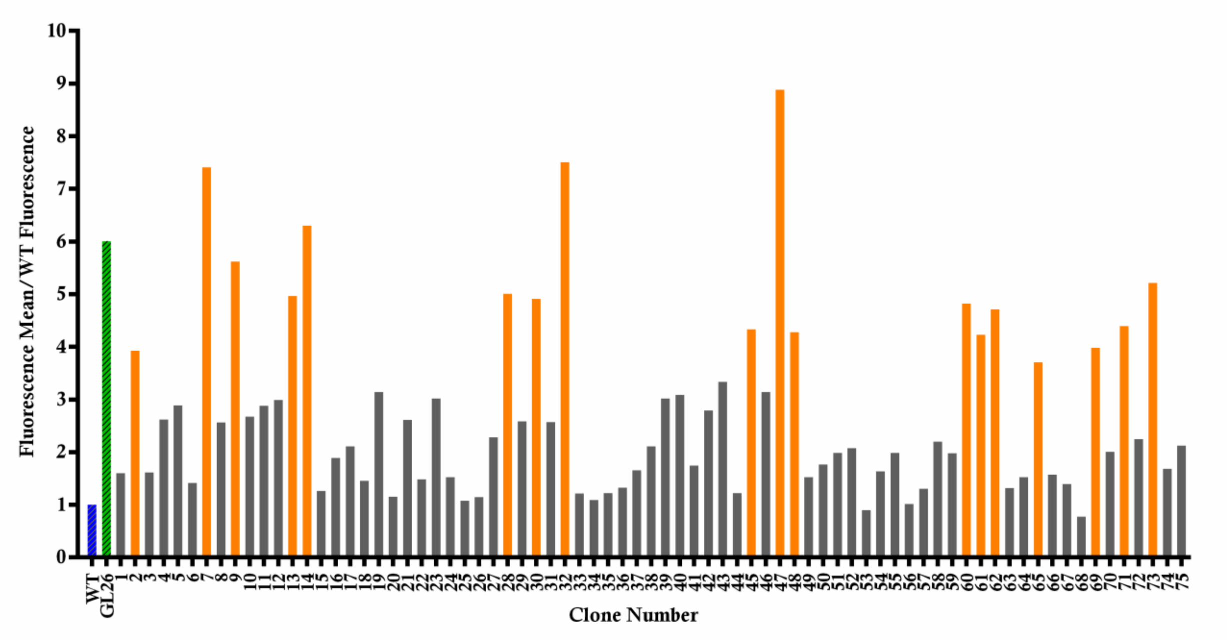

Supplementary Fig. S3. Amino acid sequence alignment of OG resistant A2aR variants retrieved by YDDS. The amino acid sequence alignment of A2aR-WT, thermostable GL26 mutant and 18 highly OG resistant A2aR variants present in the YDDS library was generated by using T-Coffee multiple sequence alignment server: http://tcoffee.crg.cat/apps/tcoffee/do:regular. The positions of structure-stabilizing substitutions, the transmembrane helices (TMH) and the cytoplasmic helix 8 are indicated.

```
                    TMH1                              TMH2
WT      1 MPIMGSSVYITVELAIAVLAILGNVLVCWAVWLNSNLQNVTNYFVVSLAAADIAVGVLAI
GL26    1 MPIMGSSVYITVELAIAVLAILGNVLVCWAVWLNSNLQNVTNYFVVSAAAADILVGVLAI
c2      1 MPIMGSSVYITVELAIAVLAILGNVLVCWAVWLNSNLQNVTNYFVVSLAAADIAVGVLAI
c7      1 MPIMGSSVYITVELAIAVLAILGNVLVCWAVWLKSNLQNVTNYFVVSLAAADIAVGVLAI
c9      1 MPIMGSSVYITVELAIAVLAILGNVLVCWAVWLNSNLQNVINYFVVSLAAADIAVGVLAI
c13     1 MPIMGSSVYITVELAIAVLAILGNVLVCWAVWLNSNLQNVTNYFVVSLAAADIAVGVLAI
c14     1 MPIRGSSVYITVELAIAVLAILGNVLVCWAVWLNSNLQNFTNYFVVSLAAADIAVGVLAI
c28     1 MPIMGSSVYITVELAIAVLAILGNVLVCWAVWLNSNLQNVTNYFVVSLAAADIAVGVLAI
c30     1 MPIMGSSVYITVELAIAVLAILGNVLVCWAVWLNSNLQNVTNYFVVSLAAADIAVGVLAI
c32     1 MPIMGSSVYITVELAIAVLAILGNVLVCWAVWLNSNLQNVTNYFVVSLAAADIAVGVLAI
c45     1 MPIMSSSVYITVELAIAVLAILGNVLVCWAVWLNSNLQNVTNYFVVSLAAADIAVGVLAI
c47     1 MPIEGSSVYIAVELAIAVLAILGNVLVCWAVWLNSNLQNVTNYFVVSLAAADIAVGVLAI
c48     1 MPIMGSSVYITVELAIAVLAILGNVLVCWAVWLNSNLQNVTNYFVVSLAAADIAVGVLAI
c60     1 MPIMGSSVNITVELAIAVLAILGNVLVCWAVWLNSNLQNVTNYFVVSLAAADIAVGVLAI
c61     1 MPIMGSPVYITVELAIAVLAIRGNVLVCWAVWLNSNLQSVTNYFVVSLAAADIAVGVLAI
c62     1 MPYMGSSVYITVELAIAVLAILGNVLVCWAVWLNSNLQNVTNYFVVSLAAADIAVGVLAI
c65     1 MPIMGSSVYITVELAVAVLAILGNVLVCRAVWLNSNLQNVTNYFVVSLAAADIAVGVLAI
c69     1 MPIMGSSVYITVELAIAVLANLGNVLVCWAVRLNSNLQNVTNYFVVSLAAADIAVGVLAI
c71     1 MPIMGSSVFITVELAIAVLAILGNVLMCWAVWLNSNLQNVINYFVVSLAVADIAVGVLAI
c73     1 MPIMGSSVYITVELAIAVLAILGNVLVCWAVWLNSNLQNVTNYFVVSLAAADIAVGVLAI
```

```
             TMH2                  TMH3                         TMH4
WT     61 PFAITISTGFCAACHGCLFIACFVLVLTQSSIFSLLAIAIDRYIAIRIPLRYNGLVTGTR
GL2    61 PFAIAISTGFCAACHGCLFIACFVLVLTASSIFSLLAIAIDRYIAIRIPLRYNGLVTGTR
c2     61 PFAITISTGFCAACHGCLFIACFVLVLTQSSIFSLLAIAIDRYIAIRIPLRYNGLVTGTR
c7     61 PFAITISTGFCAACHGCLFIACFVLVLTQSSIFSLLAIAIDRYIAIRIPLRYNGLVTGTR
c9     61 PFAITISTGFCAACHGCLFIACFVLVLTQSSIFSLLAIAIDRYIAIRIPLRYNGLVTGTR
c13    61 PFAITISTGFCDACHGCLFIARFVLVLTQSSIFSLLAIAIDRYIAIRIPLRYNGLVTGTR
c14    61 PFAITISTGFCAACHGCLFIACFVLVLTQSSIFSLLAIAIDRYIAIRIPLRYNGLVTGTR
c28    61 PFAITISTGFCAACHGCLFIACFVLVLTQSSIFSLLAIAIDRYIAIRIPLRYNGLVTGTR
c30    61 PFAITISTGFCAACHGCLFIACFVLVLTQSSIFSLLAIAIDRFIAIRIPLRYNGLVTGTR
c32    61 PSAITISTGFCAACHGCLIIACFVLVLTQSSIFSLLAIAIDRYIAIRIPLRYNGLVTGTR
c45    61 PFAITISTGFCAACHGCLFIACFVLVLTQSSIFSLLAIAIDRYIAIRIPLRYNGLVTGTR
c47    61 PFAITISTGFCAACHGCLFTACFVLVLTQSSIFSLLAIAIDRYIAIRIPLRYNGLVNGTR
c48    61 PFAITISTGFCAACHGCLFIACFVLVLTQSSIFSLLAIAIDRYIAIRIPLRYNGLVTGTR
c60    61 PFAITISTGFCAACHGCLLIACFVLVLTQSSIFCLLAVAIDRYIAIRIPLRYNGLVTGTR
c61    61 PFAITISAGSCAACHGCLFIACFVLVRTQSSIFSLLAIAIDRYIAIRIPLRYNGLETGTR
c62    61 PFAITISTGFCAACHGCLYIACFVLVLTQSSIFSLQAIAIDRYIAIRIPLRYNGLESGTR
c65    61 PFAITISTGFCAACHGCLFIACLVLVLTQSSIFSLLAIAIDRYIAIRIPLRYNGLVTGTR
c69    61 PFAITISTGFCAACHGCLFIACFVLVPSQSSIFSLLAIAIDRDIAIRIPLWYNGLVTGTR
c71    61 SFAITISTGFCAACHGCLSNACFVLVLTQCSIYSLLAFAIDRYIAIRIPLRYNGLVTGTR
c73    61 PFAITISTGFCAACHGCLFIACFVLVLTQSSIFSLLAIAIDRYIAIRIPLRYNGLVTGTR
```

```
                TMH4                                                  TMH5
WT    121 AKGIIAICWVLSFAIGLTPMLGWNNCGQPKEGKNHSQGCGEGQVACLFEDVVPMNYMVYF
GL26  121 AKGIIAICWVLSFAIGLTPMLGWNNCGQPKEGKNHSQGCGEGQVACLFEDVVPMNYMVYF
c2    121 AKGIIAICWVLSFAIGLTPMLGWNNCGQPKEGKNHSQGCGEGQVACLFEDVVPMNYMVYF
c7    121 AKGIIAICWVLSFAIGLTPMLGWNNCGQPKEGKNHSQGCGEGQVACLFEDVVPMNYMVYF
c9    121 AKGIIAICWVLSFAIGLTPMLGWNNCGQPKEGKNHSQGCGEGQVACLFKDVVPMNYMVYF
c13   121 AKGIIAICWVLSFAIGLTPMLGWNNCGQPKEGKNHSQGCGEGQVACLFEDVVPMNYMVYF
c14   121 AKGIIAICWVLSFAIGLTPMLGWNNCGQPKEGKNHSQGCGEGQVACLFEDVVPMNYMVYF
c28   121 AKGIIAICWVLSFAIGLTPMLGWNNCDQPKEGKNHSQGCGEGQVACLFEDVVPMNYMVYF
c30   121 AKGIIAICWVLSFAIGLTPMLGWNNCGQPKEGKNHSQGCGEGQVACLFEDVVPMNYMVYF
c32   121 AKGIIAICWVLSFAIGLTPMLGWNNCGQPKEGKNHSQGCGEGQVACLFEDVVPMNYMVYF
c45   121 AKGIIAICWVLSFAIGLTPMLGWNNCGQPKEGKNHSQGCGEGQVACLFEDVVPMNYMVYF
c47   121 AKGIIAICWVLSFAIGLTPMLGWNNCGQPKEGKNHSQGCGEGQVACLFEDVVPMNYMVYF
c48   121 AKGIIAICWVLSFAIGLTPMLGWNNCGQPKEGKNHSQGCGEGQVACLFEDVVPMNYMVYF
c60   121 AKGIIAICWVLSFAIGLTPMLGWNNCGQPKEGKNHSQGCGESQVACLFEDVVPMNYMVYF
c61   121 AKGIIAICWVLSFAIGLTPMLGWNNCGQPKEGKNHSQGCGEGQVACLFEDVVPMNYMVYF
c62   121 AEGIIAICWVLSFAIGLTPMLGWNNCGQPKEGKNHSQGCGEGQEACLFEVVVPMNYMVYF
c65   121 AKGNIAFCWVLSFAIGLTPMLGWNNCGQPKEGKNHSQGCGEGQVACLIEDVVSMNYMVYF
c69   121 AKGIIAICWVLSFAIGLTPMLGWNNCGQPKEGKNHSQGCGEGQVACLFEDVVPMNYMVYL
c71   121 AKGINAICWVLSFAIGLTPMLGWNNCGQPKEGKNHSQGCGEGQEACLYEDVVPMNYMVYF
c73   121 AKGIIAICWVLSFAIGLTPMLGWNNCGQPKEGKNHSQGCGEGQVACLFEDVVPMNYMVYF
```

```
                        TMH5                                TMH6
WT    181 NFFACVLVPLLLMLGVYLRIFLAARRQLKQMESQPLPGERARSTLQKEVHAAKSLAIIVG
GL2   181 NFFACVLVPLLLMLGVYLRIFLAARRQLKQMESQPLPGERARSTLQKEVHAAKSLAIIVG
c2    181 NFYACVLVPLLLMLGVYLRIFLAARRQLKQMERQPLPGERARSTLQKEVHAAKSLAIIVG
c7    181 NFFACVLVPLLLMLGVYLRIFLAARRQLKQMESQPLPGERARSTLQKEVHAAKSLAIIVG
c9    181 NFFACVLVPLLLMLGVYLRIFLAARRQLKQMESQPLPGERARSTLQKEVHAAKSLAIIVG
c13   181 NFFACVLVPLLIMLGVYLRIFLAARRQLKQMESQPLPGERARSTLQKEVHAAKSLAIIVG
c14   181 NFFACVLVPLLLMLGVYLRIFLAARRQLKQMESQPLPGERARSTLQKEVHAAKSLAIIVG
c28   181 NFFACVLVPLLLMLGVYLRIFLAARRQLKQMESQPLPGERAWSTLQKEVHAAKSLAIIVG
c30   181 NFFACVLVPLLLMLGVYLRIFLAARRQLKQMESQPLPGERARSTLQKEVHAAKSLAIIVG
c32   181 NFFACVLVPLLLMLGVYLRIFLAARRQLKQMESQPLPGERARSTLQKEVHAAKSLAIIVG
c45   181 NFFACVLVPLLLMMGVYLRIFLAARRQLKQMESQPLPGERARSTLQKEVHAAKSLAIIVE
c47   181 NFFACVLVPLLLMLGVYLRIFLAARRQLKQMESQPLPGERARSTLQKEVHAAKSLAIIVG
c48   181 NFFACVLVPLLLMLGVYLRIFLAARRQLKQMESQPLPGERARSTLQKEVHAAKSLAIIVG
c60   181 NFFACVLVPLLLMLGVYLRIFLAARRQLKQMESQPLPGERARSTLQKEVHAAKSLAIIVG
c61   181 NFFACVLVPLLLMLGVYLRIFLAARRQLKQMESQPLPGERARSTLQKEVHAAKSLAIIVG
c62   181 DFFACVRVPLLLMLGVYLRIFLAARRQLKQIESQPLPWERARSTLQKEVHAAKSLAIIVG
c65   181 NFFACVLVPLQLMLGVFLRICLAARRQLKQMESQPLPGERARSTLQKEVHAAKSLAIIVG
c69   181 NFFACVLVPLLLMLGVYLRIFLAARRQLKQMESQPLPGERARSTLQKEVHAAKSLAIIVG
c71   181 NFFACVLVPLLLMLGVYLRIFLAARRQLKQMESQPLPGERARSTLQKEVHAAKSLAIIVG
c73   181 NFFACVLVPLLLMLGVYLRIFLAARRQLKQMESQPLPGERARSTLQKEVHAAKSLAIIVG
```

```
                                  TMH6                                          TMH7           Helix-8
WT    241 LFALCWLPLHIINCFTFFCPDCSHAPLWLMYLAIVLSHTNSVVNPFIYAYRIREFRQTFR
GL26  241 LFALCWLPLHIINCFTFFCPDCSHAPLWLMYLAIVLSHTNSVVNPFIYAYRIREFRQTFR
c2    241 LFALCWLPLHIINCFTFFCPDCSHAPLWLMYLAIVLSHTNSVVNPFIYAYRIREFRQTFR
c7    241 LFALCWLPLHIINCFTFFCPDCSHAPLWLMYLAIVLSHTNSVVNPFIYAYRIREFRQTFR
c9    241 LFALCWLPLHIINCFTFFCPDCSHAPLWLMYLAIVLSHTNSVVNPFIYAYRIREFRQTFR
c13   241 LFALCWLPLHIINCFTFFCPDCSHAPLWLMYLTIVLSHTNSVVNPFIYAYRIREFRQTFR
c14   241 LFALCWLPLHIINCFTFFCPDCSHAPLWLMYLAIVLSHTNSVVNPFIYAYRIREFRQTFR
c28   241 LFALCWLPLHIINCFTFFCPDCSHAPLWLMYLAIVLSHTNSVVNPFIYAYRIREFRQTFR
c30   241 LFALCWLPLHIINCFTFFCPDCSHAPLWLMYLAIVLSHTNSDVNPFIYAYRIREFRQTFR
c32   241 LFALCWLPLHIINCFTFFCPDCSHAPLWLMYLAIVLSHTNSVVNPFIYAYRIREFRQTFR
c45   241 LFALCWLPLRIINCFTFFCPDCSHAPLWLMYLAIVLSHTNSVVNPFIYAYRIREFRQTFR
c47   241 LFALCWLPLHIINCFTIFCPDCSHAPLWLMYLAIVLSHTNSVVNPFIYAYRIREFRQTFR
c48   241 LFALCWLPLHIINCFTFFCPDCSHAPLWLMYLAIVLSHTNSVVNPFIYAYRIREFRQTFR
c60   241 LFALCWLPLHIINCFIFFCPDCGHAPLWLMYLAIVLSHTDSVVNPFTYAYRIREFRQTFR
c61   241 LFALCWLPLHIINCFTFFCPDCSHAPLWLMYLAIVLSHTNSVVNPFIYAYRICEFRQTFR
c62   241 LFALCWPPLHIINCFTLFCPDCSHAPLWLMYLAIVLSHTNSVVNPFIYAYRNREFRQTFR
c65   241 LFALCWLPLHIINCFTFFCPDCSHAPLWLMYLAIVLSHTNSVVNPFIYAYRIREFRQTFR
c69   241 LFALCWLPLHIINCFTFFCPDCSHAPLWLMYLAIVLSHTNSVVNPFIYAYRIREFRQTFR
c71   241 LFALCWLPLHIINCFTFFCPDCSHAPLWLMYLDIVLSHTNSVVNPFIYAYRIREFRQTFR
c73   241 LFALCWLPLHIINCFTFFCPDCSHAPLWLMYLAIVLSHTNSVVNPFIYAYRIREFRQTFR
```

```
                Helix-8
WT    301 KIIRSHVLRQQEPFKAAGTSARVLAAHGSDGEQVSLRLNGHPPGVWANGSAPHPERRPNG
GL26  301 KIIRSHVLRQQEPFKAAGTSARVLAAHGSDGEQVSLRLNGHPPGVWANGSAPHPERRPNG
c2    301 KIIRSHVLRQQEPFKAAGTSARVLAAHGSDGEQVSLRLNGHPPGVWANGSAPHPERRPNG
c7    301 KIIRSHVLRQQEPFKAAGTSARVLAAHGSDGEQVSLRLNGHPPGVWANGSAPHPERRPNG
c9    301 KIIRSHVLRQQEPFKAAGTSARVLAAHGSDGEQVSLRLNGHPPGVWANGSAPHPERRPNG
c13   301 KIIRSHVLRQQEPFKAAGTSARVLAAHGSDGEQVSLRLNGHPPGVWANGSAPHPERRPNG
c14   301 KIIRSHVLRQQEPFKAAGTSARVLAAHGSDGEQVSLRLNGHPPGVWANGSAPHPERRPNG
c28   301 KIIRSHVLRQQEPFKAADTSARVLAAHGSDGEQVSLRLNGHPPGVWANGSAPHPERRPNG
c30   301 KIIRSHVLRQQEPFKAAGTSARVLAAHGSDGEQVSLRLNGHPPGVWANGSARHPERRPNG
c32   301 KIIRGHVLRQQEPFKAAGTSARVLAAHGSDGEQVSLRLNGHPPGVWANGSAPHPERRPNG
c45   301 KIIRSHVLRQQEPFKAAGTSARVLAAHGSDGEQVSLRLNGHPPGVWANGSAPHPERRPNG
c47   301 KIIRSHVLRQQEPFKAAGTSARVLAAHGRDGEQVSLRLNGHPPGVWANGSAPHPERRPNG
c48   301 KIIRSHVLRQQESFKAAGTSARVLAAHGSDGEQVSLRLNGHPPGVWANGSAPHAERRPNG
c60   301 KIIRSHVLMQQEPFKAAGTSARVLAAHGSDGEQVSLRLNGHPPGVWANGSAPHPERRPNG
c61   301 KIIRSHVLRQQEPFKAAGTSARFSAAHGSDGEQVSLRLNGHPPGVWANGSAPLPERRPNG
c62   301 KIICSHVLRQQEPFKAAGSSARVLAAHGSDGEQVSLRLNGHPPGVWANGCAPHPERRPNG
c65   301 KIIRSLVLRQHEPFKAAGTSARVLAAHGSDGEQVSLRLNGHPPGVWANGSAPHPERRPNG
c69   301 KIIRSHVLRQQEPFKAAGTSARVLAAHGSDGEQVSLRLNGHPPGVWANGSAPHPERRPNG
c71   301 KIIRSHVLRQQEPFKAAGTSARVLAAHGSDGEQVSLRLNGHPPGVWANGSAPHPERRPNG
c73   301 KIIRSHVLRQQEPFKAAGTSARVLAAHGSDGEQVSLRLNGHPPGVWVNGSAPHPERRPNG
```

```
WT    361 YALGLVSGGSAQESQGNTGLPDVELLSHELKGVCPEPPGLDDPLAQDGAGVS
GL26  361 YALGLVSGGSAQESQGNTGLPDVELLSHELKGVCPEPPGLDDPLAQDGAGVS
c2    361 YALGLVSGGSAQESQGNTGLPDVELLSHELKGVCPEPPGLDDPLAQDGAGVS
c7    361 YALGLVSGGSAQESQGNTGLPDVELLSHELKGVCPEPPGLDDPLAQDGAGVS
c9    361 YAQGLVSGGSAQESQGNTGLPDVELLSHELKGVCPEPPGLVDPLAQDGAGVS
c13   361 YALGLVSGGSAPESQGNTGLPDVELLSHELKGVCPEPPGLDDPLAQDGAGVS
c14   361 YALGLVSGGSAQESQGNTCLPDVELLSHELKGVCPEPPGLDDPLAQDGAGVS
c28   361 YALGLVSGGSAQESQVNTGLPDVELLSHELKGVCPEPPGLDDPLAQDGAGVS
c30   361 YALWLVSGGSAQESQGNTGLPDVELLSHELKGVCPEPPGLDDPLAQDGAGVS
c32   361 YALGLVSGGSAQESQGNTGLPDVELLSHELKGVCPEPPGLDDPLAQDGAGVS
c45   361 YALGLVSGGSAQESQGNTGLPDVELLSHELKGVCPEPPGLDDPLAQDGAGVS
c47   361 YALGLVSGGSAQESQGNTGLPDVELLSHELKGVCPEPPGLVDPLAQDGAGVS
c48   361 YALGLVSGGSAQESQGNTGLPDVELLSHELKGVCPEPPGLDDPLAQDGAGVS
c60   361 YALGLASGGSAQESQGHTGLPDVELLSHELKGVCPEPPGLEDPPAQDGAGVS
c61   361 YALGQVSGGSAQESQGNMGLPDVGLLSHELKGVCPEPPGLDVPLAQDGAGVS
c62   361 YALGLVSGGSAQESQGNTGLPDVELLSHELKGVCPEPPGLDDPLAQDGAGVS
c65   361 YALGLVSGGSAQESLGNTGLPDVELLSHELKGVCPEPPGLDDPLAQDGAGVS
c69   361 YALGLVSGGSAQESQGNTGLPDVELLSHELKGVCPEPPGLDDPLAQDGAGVS
c71   361 YALGLVSGGSAQESQGNTGLPDVELLSHELKGVCPEPPGLDDPLAQDGAGAS
c73   361 YALGLVSGGSAQESQGNTGLPDVELLSHELKGMCPEPPGLDDPLAQDGAGVS
```

Supplementary Table S1. Mutations occurring in OG resistant A2aR variants retrieved by YDDS. Positions of the substitutions numbered according to the Ballesteros-Weinstein GPCR numbering scheme are indicated in parentheses. Mutation positions located in either Helix 8 or C-terminus (CT) are highlighted in blue. ICL and ECL are intra- and extra-cellular loops, respectively.

Clone No	Mutations								
GL26	L48A (2x46)	A54L (2x52)	T65A (2x62)	Q89A (3x37)					
c2	F183Y (5x45)	S213R (5x74)							
c7	N34K (1x60)								
c9	T41I (2x39)	E169K (ECL2)	L363Q (CT)	D401V (CT)					
c13	A72D (23x52)	C82R (3x30)	L192I (5x53)	A273T (7x37)	Q372P (CT)				
c14	M4R (1x30)	V40F (2x38)	G379C (CT)						
c28	G147D (ICL2)	R222W (6x24)	G318D (CT)	G376V (CT)					
c30	Y103F (3x51)	V282D (7x47)	P352R (CT)	G364W (CT)					
c32	F62S (2x59)	F79I (3x27)	S305G (8x60)						
c45	G5S (1x31)	L194M (5x55)	G240E (6x42)	H250R (6x52)					
c47	M4E (1x30)	T11A (1x37)	I80T (3x28)	T117N (4x38)	S329R (CT)	D401V (CT)			
c48	P313S (8x68)	P354A (CT)							
c60	F79L (3x27)	S94C (3x42)	I98V (3x46)	G162S (ECL2)	T256I (6x58)	S263G (ECL3)	N280D (7x45)	I287T (7x52)	R309M (8x64)
	V366A (CT)	N377H (CT)	D401E (CT)	L404P (CT)					
c61	S7P (1x33)	L22R (1x48)	N39S (2x37)	T68A (2x65)	F70S (23x50)	L87R (3x35)	V116E (34x57)	R293C (8x48)	V323F (CT)
	L324S (CT)	H353L (CT)	L365Q (CT)	T378M (CT)	E384G (CT)	D402V (CT)			
c62	I3Y (1x29)	F79I (3x27)	L96Q (3x44)	V116E (34x57)	T117S (4x38)	K122E (4x43)	V164E (ECL2)	D170V (ECL2)	N181D (5x43)
	L187R (5x48)	M211I (5x72)	G218W (ICL3)	L247P (6x49)	F257L (6x59)	I292N (8x47)	R304C (8x59)	T319S (CT)	S350C (CT)
c65	I16V (1x42)	W29R (1x55)	F83L (3x31)	I124N (4x45)	I127F (4x48)	F168I (45x52)	P173S (5x36)	L191Q (5x52)	Y197F (5x58)
	F201C (5x62)	H306L (8x61)	Q311H (8x66)	Q375L (CT)					
c69	I21N (1x47)	W32R (1x58)	L87P (3x35)	T88S (3x36)	Y103D (3x51)	R111W (34x52)	F180L (5x42)		
c71	Y9F (1x35)	V27M (1x53)	T41I (2x39)	A50V (2x48)	P61S (2x58)	F79S (3x27)	I80N (3x28)	S90C (3x38)	F93Y (3x41)
	I98F (3x46)	I125N (4x46)	V164E (ECL2)	F168Y (ECL2)	A273D (7x37)	V411A (CT)			
c73	A347V (CT)	V393M (CT)							
